# A Case Report of Prolonged Anaphylaxis after COVID-19 Vaccine

**DOI:** 10.5811/cpcem.2021.9.53690

**Published:** 2021-12-14

**Authors:** Lisa Armstrong, Nicole Maguire

**Affiliations:** RWJ Barnabas Health-Community Medical Center, Department of Emergency Medicine, Toms River, New Jersey

**Keywords:** COVID-19 vaccine, anaphylaxis, prolonged, case report

## Abstract

**Introduction:**

As the medical community and world have combatted the coronavirus disease 2019 (COVID-19) pandemic, a significant advance was the development of a vaccine against the virus that has already claimed over 4.5 million lives worldwide.^1^ Vaccines manufactured by Pfizer-BioNTech and Moderna were the first two COVID-19 vaccines given emergency use authorization by the United States Food and Drug Administration. Preliminary data demonstrated not only the vaccines’ efficacy rates of greater than 95% after a second dose, but also marked safety. Initial data showed only 21 cases of anaphylaxis of greater than 1.8 million doses administered. The majority of those patients had a history of anaphylaxis and presented within the first 15 minutes after administration of the vaccine.^2^

**Case Report:**

We describe a patient who had an anaphylactic reaction to her second dose of the Pfizer BioNTech severe acute respiratory syndrome coronavirus 2 (SARS CoV-2) vaccine with no prior history of allergic reactions or anaphylaxis. This reaction required multiple doses of epinephrine and a four-day hospitalization. We review both the available reports of anaphylaxis to the SAR CoV-2 vaccine and information on other prolonged cases of anaphylaxis.

**Conclusion:**

Our case report is unique in that the patient, despite no prior history of anaphylaxis, had a prolonged course requiring a four-day hospitalization. To our knowledge this is one of the first case reports of prolonged anaphylaxis after the second dose of Pfizer BioNTech COVID-19 vaccine in a patient with no history of prior anaphylaxis.^3^

## INTRODUCTION

The United States Food and Drug Administration has issued emergency use authorization for multiple severe acute respiratory syndrome coronavirus 2 (SARS CoV-2) vaccines. The first two vaccines, Pfizer BioNTech (Mainz, Germany) and Moderna (Moderna, Inc, Cambridge, MA), use messenger ribonucleic acid (mRNA) nanotechnology with a lipid-based nanoparticle base. These vaccines were considered by many to be a sign of hope amidst this global pandemic. While this is new technology, preliminary studies have shown it to be both effective with efficacy rates of greater than 95% and safe. Although the rates of anaphylaxis with mRNA vaccines are relatively low (4.7 cases per million doses), it is still believed to be tenfold higher than of prior vaccines.^3^,^4^

Anaphylaxis is a life-threatening condition that can occur after exposure to any medication, food, vaccine, or environmental trigger. It involves multiple systems including airway, skin, respiratory, and gastrointestinal tract, often presenting with a rash, nausea, shortness of breath, and in severe cases airway swelling. With the SARS CoV-2 vaccine, the majority of cases have presented after administration of the first dose, often within 30 minutes, with 75% of patients presenting in the first 15 minutes. Interestingly, 78% percent of cases occurred after the first dose, and the majority (77% percent) had a history of anaphylaxis. The majority (92%) of cases were treated with epinephrine, and 11% required intubation. While 48% required hospitalization, the average length of stay was 1–3 days.^3^ The most recent guidelines have cautioned against vaccination in those with a history of anaphylaxis, especially to medications or vaccines, as the majority of cases of anaphylaxis after vaccination have occurred in patients with a prior history.^5^

We present a case of a 43-year-old woman with no prior history of anaphylaxis, who had a prolonged anaphylactic reaction to her second Pfizer BioNTech coronavirus disease 2019 (COVID-19) vaccine, requiring a four-day hospital stay. The case is unique not only because the patient had no documented history of anaphylaxis, but also due to the length of symptoms and protracted course.

## CASE REPORT

The patient was a 43-year-old female with no significant past medical history. She received her first Pfizer BioNTech SARS CoV-2 vaccine without incident, and only reported mild fatigue and myalgias afterwards. After she received her second dose of the Pfizer BioNTech COVID-19 vaccine, at the end of her 15-minute observation period she reported a tingling sensation on the back of her tongue. After a short period of further observation and continued symptoms she was brought to the emergency department (ED). On arrival to the ED, she had normal vital signs and no abnormality on physical exam. She was offered steroids and diphenhydramine but declined. Within 15 minutes, she began to cough and complained of tightness in her throat.

On re-evaluation, the patient was noted to have wheezing and edema of her uvula. She was given diphenhydramine, methylprednisolone, and famotidine intravenously (IV), along with IV fluids and albuterol inhaler. After five minutes her symptoms progressed, and she began to vomit, reported worsening throat swelling, and was tachycardic with a heart rate of 150 beats per minute. She was then treated with epinephrine 0.3 milligrams intramuscular (IM) to her deltoid area. However, she continued to wheeze, had uvular swelling, was unable to talk (due to throat swelling), and was noted to be pale, with continued tachycardia. A second dose of epinephrine was given IM (to her deltoid), and within five minutes her wheezing and vomiting resolved, she was able to talk, and reported improvement in throat swelling. She was kept in the ED for two hours of observation, with a continued hoarse voice, subjective tightness in her throat, and noted uvular swelling on exam. Due to the persistence of swelling, she was placed in the observation unit for continued monitoring.

Several hours after arrival to the observation unit, the patient again rapidly developed increased throat tightness, vomiting, and shortness of breath, with wheezing and uvular swelling on exam. She was given additional methylprednisolone and diphenhydramine IV, along with a racemic epinephrine nebulizer and albuterol inhaler. When her symptoms did not improve, she was given a third epinephrine 0.3 milligrams IM (to her thigh). She was transferred to the intensive care unit for further monitoring; however, steroids and diphenhydramine were not further administered that night. She awoke in the morning hospital day (HD) #1, with subjective tightness in her throat, a hoarse voice, and was again noted to have uvular swelling. Later that morning, she began coughing, vomiting, and noted a fine macular rash to her forearms. Physical examination again showed wheezing with increased uvular swelling, along with tachycardia. She was started on diphenhydramine IV every eight hours, methylprednisolone IV every 12 hours, racemic epinephrine nebulizers every four hours, and albuterol inhaler every four hours. She received three racemic epinephrine nebulizers that day due to cough and throat tightness.

**CPC-EM Capsule**

What do we already know about this clinical entity?

*The SARS-CoV-2 vaccine has been shown to be very safe. Most anaphylactic reactions had a history of prior reactions, unlike our patient who had no history of this*.

What makes this presentation of disease reportable?

*Our case is unique as it was to the second dose, in a patient with no prior history of anaphylaxis. It is also unusual due to the length of the reaction*.

What is the major learning point?

*Patients should be monitored for signs of anaphylaxis, even with no prior history. In addition, clinicians should be awre of the possibility of rebound reactions*.

How might this improve emergency medicine practice?

*Clinicians can counsel patients on the risk of anaphylaxis to this vaccine, and raise awareness of the dangers of rebound and prolonged anaphylactic reactions*.

The following morning (HD# 2) she was noted to have a diffuse, pruritic macular rash to her chest, arms, and face, and uvular edema was still appreciated. She had two episodes of coughing, throat tightness, and wheezing requiring additional racemic epinephrine nebulizers and diphenhydramine IV. Her methylprednisolone was increased to every eight hours after these episodes, and she was transferred to the telemetry floor. On HD# 3 her cough and wheezing had resolved; however, her rash, sensation of throat swelling, and uvular swelling persisted. Only one racemic epinephrine nebulizer was given on that day. On HD# 4 wheezing had resolved; however, rash, throat tightness, and hoarse voice continued. Pulmonary consult recommended trial to oral steroids with taper on discharge.

The patient was discharged home on HD# 4 with an epinephrine auto-injector, famotidine, and a prednisone taper. Rash was still present at discharge and did not resolve until two days after discharge. She was seen by her primary care physician (PCP) one week after the initial incident for follow-up, still complaining of throat tightness and a hoarse voice. The PCP visualized minimal edema of the uvula. The following day, she saw an otolaryngologist who performed laryngoscopy; no laryngeal swelling was noted. He recommended continuing famotidine and prednisone, and added a nasal antihistamine. The patient was seen by an allergist three weeks after her initial reaction. Skin tests for polyethylene glycol 3350 (using Miralax) and polysorbate 80 (using Refresh eye drops) were non-reactive. Patch testing was also performed, and negative as well. She was advised to continue to carry an epinephrine auto-injector. She has not had any further reactions to date.

## DISCUSSION

Anaphylaxis is a broad term given to a wide complex of symptoms, often presenting with quickly escalating respiratory or cardiovascular collapse. Less commonly, biphasic reactions can occur, with a recurrence of symptoms most commonly within three to four hours, rarely as late as 12–24 hours after the initial occurrence. Protracted anaphylaxis is even more rare and can present as persistent hypotension lasting for several days. The majority of patients with biphasic reactions presented with the same symptoms as their initial presentation.^6^ Patients who presented greater than 30 minutes after exposure were 2.8 times as likely to have a protracted course. Our patient presented with signs of an allergic reaction within 15 minutes but did not devolve into anaphylaxis until almost 30 minutes had elapsed. While she did not develop hypotension, she was noted to have persistent mucous membrane swelling, wheezing, throat tightness, hoarse voice, and a pruritic rash. Her symptoms lasted for almost a week after her initial exposure, which is unusual. While our patient had multiple episodes it is unlikely that this was truly a biphasic reaction, as her symptoms never completely resolved during this time.

It is important that patients be carefully monitored after anaphylaxis for the potential of a second phase or protracted reaction. Failure to identify and correctly treat can lead to recurrent reaction, with potentially fatal consequences.^7^ While epinephrine is a mainstay of treatment, other medications are often necessary, including antihistamines and/or glucocorticoids. It is possible that our patient’s recurrence and prolonged course was related to glucocorticoids and antihistamines being held the night of her admission, and then when restarted were at a relatively low dose with twice daily dosing. This theory is somewhat supported as her symptoms improved once she received both medications every eight hours. Prolonged observation (eight to 24 hours) should be recommended in cases with a slow onset of symptoms resulting in severe anaphylaxis, or those with the risk of continued exposure of allergen due to the risk of recurrent reactions.^8^ This would apply to our patient, as she had a delayed presentation, and the IM administration of a vaccine should raise concern for continued exposure.

The Vaccine Adverse Event Reporting System is used in the United States to monitor adverse reactions, including anaphylaxis to the SARS-CoV-2 vaccines. While 175 cases of severe allergic reactions had been reported, only 21 were determined to be anaphylaxis. The majority experienced symptoms within 15 minutes, and 90% were treated with epinephrine. While 90% of cases were treated in the ED, only 20% required hospitalization. Fortunately, 95% of cases reported recovering with no adverse effects, and there were no reported deaths. The median age was 43, and 81% had a history of prior allergic reaction. Interestingly, 90% of anaphylaxis occurred in women, but this may be related to a higher percentage of women receiving the vaccine.^2^ Our 43-year-old patient had no history of anaphylaxis and had several episodes of recurrent symptoms during her four-day hospital stay.

## CONCLUSION

The development of vaccines against SARS-CoV-2 has been a source of hope in the global pandemic. While the vaccine was rapidly developed, studies have demonstrated not only is it efficacious in preventing death and severe disease, but it is also extremely safe. Despite this there have been several reported episodes of anaphylaxis. The majority of those patients had a history of anaphylaxis and developed symptoms shortly after vaccine administration. While most cases of anaphylaxis were treated with epinephrine, the vast majority were able to be discharged home. Our case is unique in that it occurred in a patient with no known history of anaphylaxis, who had a protracted case requiring a four-day hospitalization. Fortunately, our patient recovered, and has had no long-term complications from this event.
